# Diet of the Dingo in Subtropical Australian Forests: Are Small, Threatened Macropods at Risk?

**DOI:** 10.3390/ani13142257

**Published:** 2023-07-10

**Authors:** Dusty McLean, Ross Goldingay, Mike Letnic

**Affiliations:** 1 Faculty of Science and Engineering, Southern Cross University, Lismore, NSW 2480, Australia; ross.goldingay@scu.edu.au; 2 Centre for Ecosystem Science, School of Biological, Earth and Environmental Sciences, University of New South Wales, Sydney, NSW 2052, Australia; m.letnic@unsw.edu.au

**Keywords:** dingo, carnivore diet, apex predators, prey availability, seasonal variation, threatened species, long-nosed potoroo, red-legged pademelon, black-striped wallaby, koala

## Abstract

**Simple Summary:**

Carnivores play an important part in maintaining ecosystem health by limiting the population sizes of their prey. However, their feeding habits can also place populations of threatened species at risk of extinction or decline. We examined the diet of Australia’s largest terrestrial carnivore, the dingo, to determine whether it threatens at-risk small macropods in two subtropical forests. We found that although dingoes may prey upon some threatened macropods, they do not appear to do so at rates that will affect population persistence. We show that dingoes in some subtropical Australian forests generally target prey according to availability but also according to accessibility. Our study suggests that at present, dingoes do not appear to pose a threat to threatened macropods or some other threatened mammals in either of the forests surveyed.

**Abstract:**

Carnivores fulfil important ecological roles in natural systems yet can also jeopardise the persistence of threatened species. Understanding their diet is, therefore, essential for managing populations of carnivores, as well as those of their prey. This study was designed to better understand the diet of an Australian apex predator, the dingo, and determine whether it poses a threat to at-risk small macropods in two floristically different yet geographically close reserves in subtropical Australia. Based on an analysis of 512 scats, dingo diets comprised 34 different prey taxa, of which 50% were common between reserves. Our findings add support to the paradigm that dingoes are opportunistic and generalist predators that prey primarily on abundant mammalian fauna. Their diets in the Border Ranges were dominated by possum species (frequency of occurrence (FOC) = 92.5%), while their diets in Richmond Range were characterised by a high prevalence of pademelon species (FOC = 46.9%). Medium-sized mammals were the most important dietary items in both reserves and across all seasons. The dietary frequency of medium-sized mammals was generally related to their availability (indexed by camera trapping); however, the avoidance of some species with high availability indicates that prey accessibility may also be important in dictating their dietary choices. Other prey categories were supplementary to diets and varied in importance according to seasonal changes in their availability. The diets included two threatened macropods, the red-legged pademelon and black-striped wallaby. Our availability estimates, together with earlier dietary studies spanning 30 years, suggest that the red-legged pademelon is resilient to the observed predation. The black-striped wallaby occurred in only two dingo scats collected from Richmond Range and was not detected by cameras so the threat to this species could not be determined. Two locally abundant but highly threatened species (the koala and long-nosed potoroo) were not detected in the dingoes’ diets, suggesting dingoes do not at present pose a threat to these populations. Our study highlights the importance of site-based assessments, population monitoring and including data on prey availability in dietary investigations.

## 1. Introduction

Predation by mammalian carnivores (hereafter carnivores) can jeopardise the persistence of threatened species [1,2,3,4,5]. Population declines [6,7], reintroduction failures [8,9,10], and extinctions of threatened species [11,12] have been attributed to predation by carnivores around the world, including wolves, cats, foxes, and dingoes. In the face of global biodiversity loss, identifying and mitigating threats likely to contribute to further species declines and losses are conservation priorities [13,14,15]. Where species are threatened by predation, carnivores are often controlled by means of destruction or exclusion [5,9,12,16,17,18].

Whilst carnivores can indeed possess the latent propensity to threaten species, they also perform critical regulatory functions within ecosystems by limiting the population sizes of their prey and, in the case of top-order or apex carnivores, that of smaller predators [19,20,21]. Consequently, the effects of carnivore removal may cascade through trophic levels and disrupt an ecosystem’s structure [22,23,24,25,26], the outcomes of which may prove detrimental to species and the ecosystem at large [27,28,29,30,31]. To avoid ecological degradation, the management of carnivores must consider their role within a given ecosystem and the potential implications of their control [20,21,23,32].

The dingo (*Canis dingo* [33,34] or *Canis familiaris* [35,36]—its taxonomy is disputed [37,38,39]), Australia’s largest terrestrial carnivore, is one species warranting further investigation into its ecological role. Dingoes have long been controlled due to conflicts with livestock producers [40,41] and are known to prey upon a range of threatened species [42,43,44,45,46,47]. In some cases, they have also compromised conservation efforts [10,48]. However, as apex predators, dingoes provide important regulatory services to ecosystems and can suppress populations of their prey, including ecologically and economically damaging introduced (e.g., rabbits, goats) and native herbivores (e.g., kangaroos) [45,49,50,51,52,53]. In parts of their range, they may also suppress feral cats and foxes, the invasive mesopredators responsible for most of Australia’s mammal extinctions and declines [29,54,55,56,57,58,59,60,61], though evidence for this is variable and often contested [46,62,63,64,65,66]. Considered a threat to biodiversity by some [47] and keystone species by others [54,67,68], monitoring dingoes’ diets is critical to both their management and that of threatened species.

By analysing dingo scats, past studies have revealed broadly applicable dietary trends, including that dingoes are opportunistic and generalist predators, which tend to select for abundant mammalian prey [69]. However, because of their ecological plasticity, dingoes, like other widespread carnivores such as the Eurasian lynx (*Lynx lynx*) [70], the grey wolf (*Canis lupus*) [71], and the puma (*Puma concolor*) [72], have highly variable diets that are influenced by spatial and temporal variation in prey species assemblages and availability [10,45,69,73,74,75]. As dingo diets are subject to change, the outcomes of dietary studies are best viewed as a snapshot of a given population’s feeding habits at a particular point in time. Accordingly, the presence or absence of a species of concern in a particular diet may not alone provide an accurate indication of predation risk [43], and thus there can be immense value in conducting dietary studies at locations with a history of such studies.

Dingoes are abundant in subtropical North-eastern New South Wales (NE NSW), a region with several threatened macropodoid species (hereafter macropods), including the long-nosed potoroo (*Potorous tridactylus*), red-legged pademelon (*Thylogale stigmatica*), and black-striped wallaby (*Notamacropus dorsalis*) [76,77]. An important aspect of understanding dingoes’ diets is considering whether predation by dingoes may threaten already threatened species [43,47]. As dingoes are known predators of threatened macropods [48,73,78,79,80,81] and prey predominantly upon medium-sized mammals in subtropical regions [69], there is concern regarding whether these macropods are threatened by dingo predation. Two dietary studies [82,83] have been conducted in this region, both focussing heavily on Richmond Range National Park. These studies identified pademelons as important dietary components but found no evidence of consumption by dingoes on long-nosed potoroos or black-striped wallabies, which are less abundant [76]. However, both studies have limitations that constrain their ability to truly represent dingo diets in NE NSW: Barker et al. [82] sampled over just one month, and Glen et al. [83] had a very small sample size. Given these limitations and the knowledge that dingo diets may change over time, there is a need for a contemporary and robust analysis of dingo diets in NE NSW.

Our study aimed to investigate the diets of dingoes and relate this to the availability of prey (based on camera trapping) in two floristically different reserves in subtropical Australia: the Border Ranges National Park and Richmond Range National Park. Both reserves contain populations of several threatened mammals that are known prey of dingoes. The diet of dingoes in Richmond Range has been described twice in the last 30 years [82,83], providing the opportunity to investigate whether their diet has changed over time (e.g., Lunney et al. [73]) and whether there is any evidence that some threatened mammals are at risk of continuing to decline due to dingo predation.

## 2. Materials and Methods

### 2.1. Study Areas

North-eastern NSW experiences a largely subtropical climate and annual rainfall greater than 1000 mm. The region is renowned for its high biodiversity, mountain ranges, and ancient Gondwanan rainforest communities. Situated on the border between NSW and Queensland, the Border Ranges (−28.379, 153.085) features dense subtropical rainforest at mid-elevations (750–950 m) and cool temperate rainforest dominated by Antarctic beech (*Nothofagus moorei*) at higher elevations (950–1100 m). Richmond Range (−28.797, 152.739) lies at lower elevations (200–600 m), approximately 60 km to the southwest. It supports a diverse range of vegetation communities, including dry sclerophyll forests dominated by Richmond Range spotted gum (*Corymbia variegata*), wet sclerophyll forests dominated by flooded gums (*Eucalyptus grandis*), and subtropical rainforest characterised by sparse ground cover and *Ficus* spp. Both reserves support populations of long-nosed potoroos and red-legged pademelons; however, black-striped wallabies occur only in restricted sections of Richmond Range.

### 2.2. Scat Collection

Dingo scats were collected along the Brindle Creek Road loop section of the Border Ranges and within the southern half of Richmond Range on a seasonal basis from spring 2020 to winter 2022. Extended periods of severe wet weather, reserve closures, and COVID-19 complications prevented the collection of Richmond Range’s winter 2021 sample and the summer and autumn 2022 samples from both reserves. Because of this, samples were pooled into seasonal groups for each reserve, and any effects associated with year of collection were not investigated. Roads and tracks within the two reserves were checked towards the end of each season, so unless scats were highly aged and degraded, they were considered representative of that season and valid for seasonal analysis.

Scat pieces within 1 m^2^ were considered to represent one sample unless obvious differences in colour, moisture content, or smell indicated the presence of more than one sample. Scat samples were sent to an expert analyst (Georgeanna Story—Scats About Ecological, e.g., cited in Augusteyn et al. [10], Doherty et al. [69], Cupples et al. [84], and Letnic et al. [85]) to identify prey species from hair and bone fragments found within the scat samples. Remains within scats were identified to species level or the next lowest taxonomic level achievable. We recognise that while the term ‘prey’ is used, scats may also have contained items scavenged from carrion or ingested from plants.

### 2.3. Prey Availability Assessments

Scat data alone may be inadequate to assess predation risk to prey [43], so camera trapping was conducted concurrently with scat collection to provide data on the availability of potential prey species. We established 40 camera trap locations along the roads and trails used for scat collection within each reserve. At each location, we tied a camera trap to a tree at a height of 40 cm and directed it towards a bait canister containing standard peanut butter and oat bait mix (see McHugh et al. [76]). This design is optimised to survey small macropods and other ground-dwelling mammals vulnerable to dingo predation [76]. To ensure site independence, we positioned the cameras at least 400 m apart from one another. This distance extends beyond the home range of long-nosed potoroos [86], red-legged pademelons [87], and other prey species, so it was unlikely that individuals would be detected at more than one camera site. Cameras were deployed in each reserve for 6–12 weeks in the winter months of 2021 and 2022. Due to logistical constraints, it was not feasible to operate cameras year-round, so we deployed cameras in winter as it represented the best compromise in detectability for our two target species, the long-nosed potoroo and red-legged pademelon [88]. Detection probabilities for the black-striped wallaby are highest in spring and intermediate in winter [89]. The habitat types preferred by the black-striped wallaby were uncommon in our immediate survey area, so we expected few detections of this species and instead focused on maximising detections of the other two species.

We used the images obtained from each deployment to create daily detection histories for mammalian prey species that were detected an average of 10 or more times across the two deployment periods in each reserve. Species that were detected infrequently may be better detected by other sampling techniques (e.g., spotlighting), so to avoid underestimating their availability, they were omitted from analyses. Species that were unable to be identified reliably (e.g., small mammals) were also omitted. We calculated each species’ relative availability (*p*) as its proportion of all species activity as follows:

*p* = (*n_i_*/*N*),

where *n_i_* represents the average daily detections of *i*th species, and *N* is the sum of all retained species’ average daily detections. This calculation assumes that a higher number of individuals would produce more daily detections and standardizes availability values across a set of prey that are well surveyed by our camera trap design.

### 2.4. Dietary Analysis

For consistency and comparability, the approaches to dietary analysis resembled those taken in similar studies [82,83,84,90,91,92,93]. We assessed dingo diet by calculating both the frequency of occurrence (i.e., prey items were recorded on a presence/absence basis, summed and expressed as a percentage of scats containing that particular item) and the percentage volume of each prey item within each scat (estimated visually by the analyst) [94]. These methods each have limitations requiring consideration. The frequency of occurrence method can introduce error by overestimating the importance of foods that are eaten often but in small amounts (e.g., insects) [92]. The percentage volume method can compensate for this but may underestimate the importance of easily digested items (i.e., matter that is readily absorbed and contributes little to the bulk of scats), though it is not reported to be a significant limitation. Because the two methods complement each other, they were both used [91,92].

Data were tabulated, and prey species were ranked according to their importance (as inferred from each method). Spearman rank correlation coefficients (r_s_) were then used to test for agreement between the two methods (values close to r_s_ = 1 indicate good agreement between methods).

We used Brillouin’s diversity index (adopted from Glen and Dickman [91]) to estimate the diversity of prey in the scats, according to the following formula:

*H* = ln *N*! − ∑ ln *n_i_*!/*N*,

where *H* is the diversity, *N* is the total number of individual prey within the scat, and *n_i_* is the number of individual prey items within the *i*th category (i.e., within each species) [95,96]. We then plotted cumulative diversity (*Hk*) against the number of scats analysed (*k*) to determine whether sample sizes were sufficient to adequately assess diet (inferred by an asymptote).

To examine whether dietary composition differed among seasonal samples, we assigned prey species within scats to one of 7 categories: small mammals (<0.5 kg), medium-sized mammals (0.5–6.9 kg), large mammals (≥7 kg), birds, reptiles, invertebrates, and plant material (including fruits, seeds, and vegetation). Macropod remains that were unable to be identified to at least genus level were excluded from analyses as they could include small or large macropods. Mammal size categories were assigned in accordance to the maximum weights listed in Menkhorst and Knight [97].

Overall differences in dingo diets between reserves and across seasons were analysed using Primer (v6) software [98]. We created Bray–Curtis resemblance matrices using presence/absence data on either prey species within each scat (reserve comparisons) or prey category data (seasonal comparisons). We then ran analysis of similarity (ANOSIM) permutation tests on the matrices to determine if diets differed between reserves and across seasons. ANOSIM tests for dissimilarity between samples that are grouped a priori (in our case by the factors of reserve and season) and returns a statistic, *R* (ranging from −1 to +1), and an associated significance level which indicates whether mean ranked dissimilarities are greater between sample groups (towards +1) or within sample groups (towards −1) [99]. In instances in which there are more than two sample groups or treatments within a factor, ANOSIM tests will return an *R*-value and associated significance level for the effect of the factor itself (termed global *R*) and also for each pair of sample groups within the factor (i.e., treatments are tested for dissimilarity against one another) [99]. In our seasonal comparisons (which were conducted independently of reserve comparisons), the factor ‘season’ was tested for an overall effect on each reserve’s diet, and each seasonal sample was compared to one another (i.e., summer vs. autumn, summer vs. winter, autumn vs. winter, etc.) to determine whether they differed. If significant differences in diet were found between reserves, we used similarity percentages breakdown (SIMPER) to reveal the relative contribution of each prey species to observed dissimilarity. Similarly, if the effect of season was significant on a reserve’s diet, we identified which seasons differed significantly from one another and then used SIMPER to determine which prey categories were driving differences. We used distance-based redundancy analysis (dbRDA) ordinations to visually represent differences between reserves and overlayed vectors (r > 0.4) representing influential species.

We used Jacob’s selectivity index [100] to explore how the relative availability of prey related to their frequency in dingoes’ diet as follows:

*D* = (*r* − *p*)/(*r* + *p* − 2*rp*),

where *r* is the frequency of occurrence of the prey species in the dingoes’ diet, and *p* is the relative availability of the prey species. Jacob’s index values (*D*) range from −1 (prey are avoided) to +1 (prey are targeted). Values near 0 indicate that prey are selected proportionate to their availability. We use the term ‘avoided’ in the sense that a prey item appeared in the dingoes’ diet less frequently than expected given its availability. In this context, the term does not refer to spatial or temporal avoidance. For the purposes of calculating Jacob’s index, pooled groups of prey that were unable to be identified to species level (e.g., *Thylogale* spp.) were assumed to comprise the species within that taxonomic level proportional to their frequency in identified scats.

## 3. Results

A total of 664 scats were collected and sent to the analyst for identification. Of these, 512 were attributed to dingoes, 130 to foxes, 14 to feral cats, and the remainder to herbivores. As our focus was on the diet of the dingoes, it was not our intention to investigate the diets of foxes or cats. There may also be uncertainty in the identification of the fox scats, as foxes are rare in our study reserves [76] and were not detected in our camera surveys. It is possible some fox scats belonged to juvenile dingoes, but to avoid ambiguity, we excluded all scats assigned to species other than dingoes from our analyses. We do not report on these scats any further except to note that some fox scats contained red-legged pademelons but no other threatened macropods or koalas.

**Figure 1:**
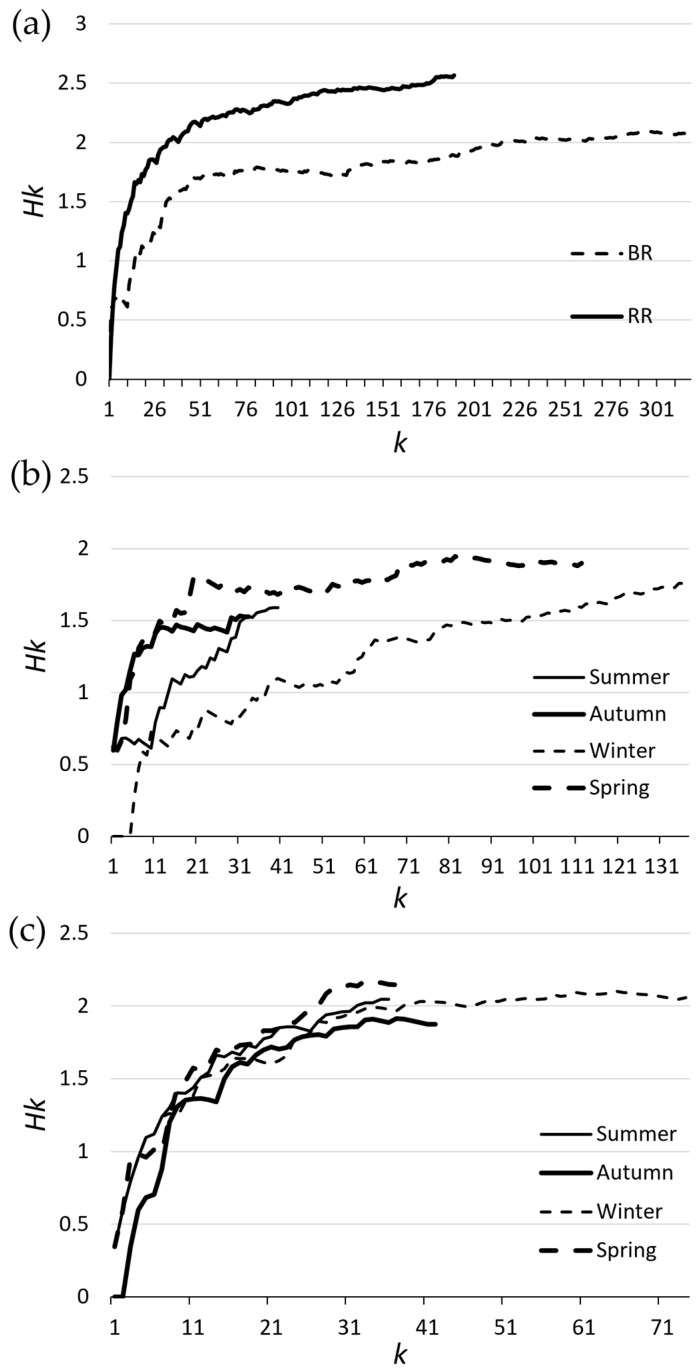
Cumulative diversity (*Hk*) of dingo diet with increasing numbers of scats (*k*) in (**a**) Border Ranges (BR) and Richmond Range (RR), (**b**) across seasons in BR and (**c**) across seasons in RR.

Of the 512 dingo scats analysed, 322 were collected in the Border Ranges and 190 were collected in Richmond Range (Table 1). The scats contained at least 34 different prey taxa, 17 (50%) of which were common between the diets in both reserves. The dietary diversity was higher in Richmond Range (29 different prey items; *H* = 2.56) than in the Border Ranges (26 different prey items; *H* = 2.07). Cumulative diversity plots indicated that, with the exception of the Border Ranges’ summer sample, the sample sizes were sufficient to adequately assess the diet in both parks (Figure 1). The Spearman rank correlations (r_s_) showed a close agreement between the frequency of occurrence (FOC) and percentage volume (Vol) analytical methods for the diets in both reserves (Border Ranges: r_s_ = 0.81, *p* < 0.01; Richmond Range: r_s_ = 0.83, *p* < 0.01).

**Figure 2:**
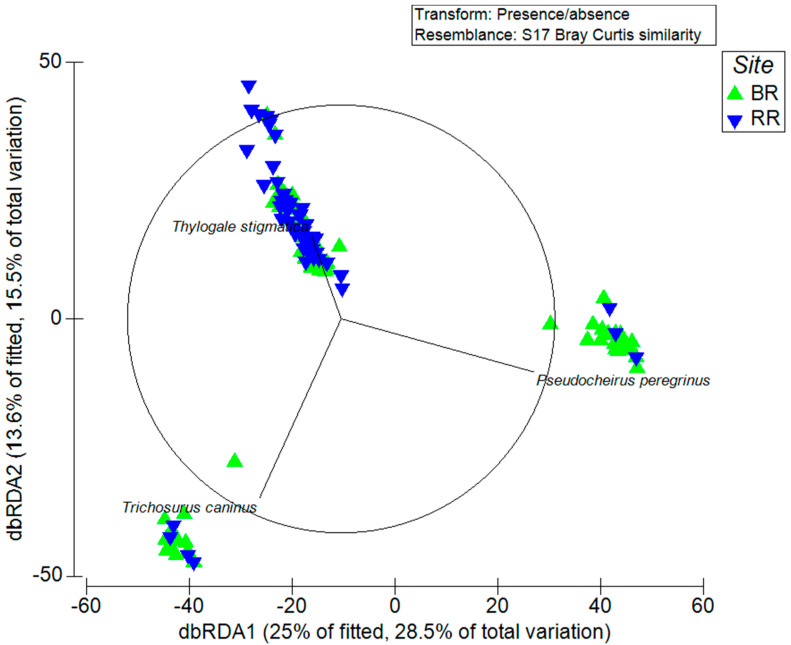
Distance-based redundancy analysis (dbRDA) ordination of scats, factored by reserve. Vectors (r > 0.4) represent influential species. BR—Border Ranges; RR—Richmond Range.

The dingoes’ diets differed significantly between the two reserves (global *R* = 0.284, *p* = 0.001). The SIMPER analysis revealed common ringtail possums (*Pseudocheirus peregrinus*), short-eared brushtail possums (*Trichosurus caninus*), and red-legged pademelons to be the most influential prey species in driving these differences (Table 2). The dbRDA plot showed a clear separation of the reserve samples, with those in the Border Ranges more likely to contain both possum species, while the samples in Richmond Range were more likely to contain red-legged pademelons (Figure 2).

### 3.1. Border Ranges

The dingoes in the Border Ranges consumed at least 26 different prey items (Table 1). Medium-sized mammals (mostly possum species) dominated their diet and accounted for 88.6% of the overall dietary volume. Birds, small mammals, plant material, and reptiles (in order of importance) comprised the remainder of the dietary volume. Common ringtail possums were the most frequently consumed prey item, followed by short-eared brushtail possums and birds. Common brushtail possums (*Trichosurus vulpecula*) were also consumed frequently and in greater volume than birds (Vol = 10.1% vs. 4.2%). Feral cat remains were found in three scats, and one scat contained the remains of the vulnerable grey-headed flying fox (*Pteropus poliocephalus*).

Five potential prey species were identified for the selectivity analyses (Table 3). Short-eared brushtail possums were selected in proportion to their availability and were the only species detected by cameras that were consumed frequently. Red-legged pademelons, despite their high availability, were detected in less than 3% of the scats. Long-nosed potoroos were not detected in the diet, and long-nosed bandicoots (*Perameles nasuta*) were highly avoided.

Season had a significant effect on the dingoes’ diet (Global *R* = 0.051, *p* < 0.01), with two of the six seasonal sample pairs differing from one another: their winter diets differed significantly from both their summer (*R* = 0.178; *p* = 0.001) and spring (*R* = 0.065; *p* = 0.001) diets (Table 2). These differences were driven by a greater occurrence of birds and plant material in their summer (birds FOC = 37.5%; plant FOC = 15%) and spring diets (birds FOC = 26.8%; plant FOC = 3.6%) than in their winter diets (birds FOC = 8%; plant FOC = 0.7%), and there were more small mammals in their winter diets (FOC = 14.6%) than in other seasons. Reptiles were more prevalent in their spring diets (FOC = 13.4%) than in any other season. The frequency of occurrence of medium-sized mammals remained consistently high across all seasons (average FOC = 95.5%); however, the dietary volume of this prey category peaked in autumn (Vol = 94.9%) and was lowest in summer (Vol = 76.1%) when the contribution of birds and plant matter was highest (Vol = 14.4% and 9.5%, respectively).

### 3.2. Richmond Range

The dingoes in Richmond Range consumed at least 29 different prey items (Table 1). Medium-sized mammals comprised the bulk of their diet, followed by large mammals, birds, plant material, and small mammals. Pademelons were the most important prey items, accounting for almost 40% of the overall dietary volume. Red-legged pademelons (FOC = 23.2%) were consumed more frequently than red-necked pademelons (*Thylogale thetis*) (FOC = 7.4%), though many scats containing pademelon remains were unable to be identified to species level (FOC = 16.3%) (Table 1). Other important dietary items included domestic cattle (*Bos taurus*), short-beaked echidnas (*Tachyglossus aculeatus*), birds, plant material (incl. figs), and unidentified macropod species. All scats containing cattle were collected in a single sampling period (winter, 2022). Black-striped wallaby remains were identified from two scats (FOC = 1.1%). Long-nosed potoroos were not detected in any scats. One scat contained rufous bettong (*Aepyprymnus rufescens*) remains, and two scats contained dingo remains, one of which was from a pup.

Estimates were made for the availability of seven potential prey species (Table 3). Red-legged pademelons were consumed in proportions close to but greater than their availability, and red-necked pademelons were selected in proportion to their availability. Long-nosed bandicoots were the most available of all prey species detected by cameras in Richmond Range yet were not detected in any scats. Short-eared brushtail possums and northern brown bandicoots (*Isoodon macrourus*) were underrepresented in the dingoes’ diet compared to their availability. Long-nosed potoroos and koalas (*Phascolarctos cinereus*) were detected frequently enough to warrant inclusion in our analyses yet were absent from the dingoes’ diet. Black-striped wallabies were not detected in any camera images.

The overall differences in the dingoes’ diets across seasons in Richmond Range were not significant (Global *R* = 0.025, *p* = 0.069). However, consistent with the results from the Border Ranges, medium-sized mammals occurred more frequently than other prey categories in all seasons (avg. FOC = 65.3%), plant matter occurred most in their summer diets (FOC = 16.7%) and reptiles occurred most in their spring diets (FOC = 2.6%). In contrast, birds and small mammals were most prevalent in their spring diets (birds FOC = 15.8%; small mammals FOC = 15.8%).

## 4. Discussion

The decline of threatened species is a well-documented and pressing issue globally [13,14,15,101]. To prevent further loss and deterioration of biodiversity in systems to which multiple potential threats exist, land managers must first identify the processes contributing to species declines. Carnivores, particularly those at the highest trophic levels, fulfil important ecological roles in natural systems yet can also jeopardise the persistence of threatened species [20,21,23,30]. Consequently, where carnivores co-occur with threatened species that are known or likely to be prey items, detailed studies are required to evaluate whether predation presents a conservation risk [43,47]. Only then can managers be appropriately informed as to whether actions aimed at controlling carnivore populations need to be implemented.

Our study investigated the diet of the dingo, an Australian apex predator, in two subtropical Australian forests. Our findings support the paradigm that dingoes are generalist carnivores that primarily prey on abundant mammalian fauna [69,74,75,81,102]. Consistent with other dietary studies of dingoes in subtropical forests, medium-sized mammals occurred most frequently and in the greatest volumes in both reserves, and prey species from other size categories supplemented their diet in varying proportions [69,82,92]. A broad range of prey items was identified; however, their diets were dominated by certain highly abundant taxa: possums in the Border Ranges and pademelons in Richmond Range.

### 4.1. Reserve Comparisons

Dietary composition varied considerably between reserves. Species in the large mammal prey category occurred only in the Richmond Range diet and mostly comprised domestic cattle and large macropod species. Whilst cattle have been identified in Richmond Range dingo diets before [82,83], this finding was notable. In the weeks prior to the winter 2022 sample collection, a number of cattle entered the reserve through a storm-damaged boundary fence. All scats containing cattle were collected during this period, which highlights the dingo’s ability to exploit variable resources (sensu Newsome et al. [102]).

Disparities in the occurrence of macropod species can mostly be attributed to differences in habitats between reserves. Some macropods found in the dingoes’ Richmond Range diet, such as black-striped wallabies, red-necked wallabies, and rufous bettongs, require grassy, open forest habitats [89,103] and are, therefore, not present in the areas sampled within the Border Ranges where this forest type is absent. Swamp wallabies have a broader ecological tolerance and occur across a range of habitats yet were also absent from the dingoes’ Border Ranges diet. The little information available on the ecology of this species in NE NSW suggests that it prefers wet and dry sclerophyll forests [104,105,106], though it is known to occur in rainforest elsewhere [107]. Historically, swamp wallabies have been important prey for dingoes in NE NSW [78,79,80,82,83]. The comparatively low dietary frequency of swamp wallabies reported here likely reflects differences in sampling locations, as the other studies were conducted several hundred kilometres south of our study areas [78,79,80], sampled a larger proportion of Richmond Range [82], or pooled scats collected from across the broader landscape [83]. Most other species accounting for dietary variations were known to be available as prey in both reserves (e.g., long-nosed bandicoots, bush rats, snakes) but were not present in the scats. That the dingoes’ diets varied between the two floristically different reserves highlights the importance of site-based assessments and cautions against generalising the results from one ecosystem across an entire landscape or bioclimatic zone.

### 4.2. Seasonal Variation in Diet

Our finding that the dingoes’ dietary composition varied across seasons in the Border Ranges marks a novel addition to the literature, because studies exploring seasonal variation in dingo diets are scant (examples include: [108,109,110,111]), and none have yet been conducted in subtropical areas. However, we found no significant seasonal influence on the dingoes’ diet in Richmond Range, but this is likely a consequence of smaller sample sizes in this reserve. In addition, availability estimates were made only for medium-sized mammals in winter, so our interpretation of the observed trends relies on published data. Future studies would benefit from obtaining larger seasonal samples and in situ seasonal availability estimates for a wider range of prey.

Seasonal variation was most profound in prey categories that were supplementary to the core diet of medium-sized mammals. Supplementary prey categories may compensate for temporary reductions in the availability of prey species that make up the core diet [109]; however, we found little evidence of this here, as the occurrence of medium-sized mammals remained consistently high across all seasons. The seasonal peaks in the dietary occurrence of supplementary prey categories generally correlated with important aspects of their life histories, so our findings instead provide further evidence that dingoes exploit periods of increased prey activity and availability [110]. For example, reptiles occurred most in the dingoes’ spring diets, presumably reflecting an increase in activity and availability associated with warming temperatures and emergence from brumation [109,110]. Other examples include a higher prevalence of plant material in the dingoes’ summer diets in both reserves and birds in the summer and spring diets of dingoes in the Border Ranges. Though birds and plant material were generally not identifiable to the species level, figs (*Ficus* spp.) and green and yellow feathers thought to belong to green catbirds (*Ailuroedus crassirostris*) were found in several scats. The increased occurrence of these species can also be attributed to their heightened availability: figs and other fruiting trees in subtropical ecosystems tend to fruit most reliably in the wet summer months [112], and bird species tend to breed in the spring and summer when resource availability is highest [112,113]. However, given our diversity curve for the Border Ranges summer sample failed to reach an asymptote (Figure 1), any interpretation of our results should consider that a greater sample size would be required to better characterize the summer diet of the dingoes in the Border Ranges.

### 4.3. Threatened Mammals

The occurrence of threatened macropods in the dingoes’ diets remains broadly consistent with that in earlier dietary studies in subtropical NE NSW. The dietary frequency of threatened red-legged pademelons (and, to a lesser extent, sympatric, red-necked pademelons) in Richmond Range closely resembled the findings of Barker et al. [82] and extends an established history of high predation by dingoes on pademelons in NE NSW to span 30 years [83]. While such prolonged and high levels of predation on a threatened species could trigger conservation concerns [10], recent research has shown pademelons to be highly abundant in Richmond Range [76], and our study has demonstrated that they are preyed upon in proportions close to their availability. The Jacob’s Index for red-legged pademelons indicated some degree of targeting; however, the score was close to zero and far from values considered high risk (*D* > 0.5) [93]. Thus, we conclude that although pademelons in Richmond Range remain important prey of dingoes, the 30-year period of high predation has not led to a population decline.

Dingoes in the Border Ranges consumed red-legged pademelons much less frequently than expected, given high availability, and instead preyed heavily on various possum species. We hypothesise that this reflects differences in hunting strategies between the dingoes in the two reserves. The structural complexity of the Border Ranges’ rainforest may hinder dingo mobility and their ability to pursue cursorial prey, such as the red-legged pademelon. This assumption is also suggested by the relative ease and higher density at which scats were located along the roads in the Border Ranges compared to those in Richmond Range where rainforest ground cover is comparatively sparse, despite the higher occupancy of dingoes in the latter reserve [76]. Possums, though generally arboreal, are vulnerable to terrestrial predators when they forage and move along the forest floor [114]. We hypothesise that the high occurrence of possum species and rarity of red-legged pademelons in the diet of dingoes in the Border Ranges is, therefore, likely a function of optimal foraging, whereby carnivores seek to optimize their net energy gain [108,115,116].

Long-nosed potoroos were not detected in the dingo diets reported here, nor in those reported by Barker et al. [82] or Glen et al. [83] during the past 30 years. In other parts of the range, the availability of this species has been linked to its occurrence in dingo diets [73,117]. For example, Lunney et al. [73] reported an increase in the occurrence of long-nosed potoroos in dingo scats over 15 years (1% to 19%), which they suggested reflected an increase in abundance with understorey-thickening post logging. Long-nosed potoroos had a higher availability in the Border Ranges than in Richmond Range (see also McHugh [88]) yet did not feature in either diet, suggesting that their availability was not associated with increased predation risk by dingoes in our study reserves. Potoroos utilise areas of dense ground cover as refuge from predators [76,118,119], a behavioural trait also shared by bandicoots [120]. Our analysis indicated that bandicoots were avoided by the dingoes, despite their high availability, which may suggest this microhabitat preference effectively reduced their predation risk. However, the use of ground cover alone may not fully explain the avoidance of these prey species reported here, as high levels of predation by dingoes on potoroos and bandicoots have been reported elsewhere [42,44,73,81,117]. These studies also reported low or reduced incidences of larger mammalian prey, so we hypothesise that the lower-than-expected dietary frequency of potoroos and bandicoots in our study may reflect diet preferences [43,117] linked to the high availability of larger and more easily accessible prey species (e.g., possums and pademelons).

Black-striped wallabies were identified from two scats in Richmond Range. These represent the first records of dingo predation on this endangered macropod in NSW. Although rare in the dingoes’ diet, the species was absent from the camera trap images, so their threat of predation is unknown. Sampling bias existed in that whilst black-striped wallabies prefer low-elevation eucalypt forest [89], the tracks along which the scats were collected and the cameras were operated in Richmond Range mostly followed wet sclerophyll and rainforest ridgelines. However, some camera locations did survey areas occupied by the black-striped wallaby, and some scat collection areas were within a 2 km vicinity of habitats occupied by this species (dingoes travel an average of 2 km between feeding and defecation [73]). Detailed data gathered from areas more heavily occupied by this species would be required to confidently assess whether it is threatened by dingoes.

Our study also provides important insight into the threat posed by the dingo to the endangered koala. Koalas are highly abundant in Richmond Range, with this reserve containing one of the largest known populations in NSW [121]. Our camera traps showed they were active on the ground and therefore exposed to the threat of dingo predation as they moved between trees, which they do on a daily basis (e.g., Marsh et al. [122]). However, this species was not detected in the dingoes’ diet. Records of koala predation by dingoes and wild dogs are almost entirely limited to isolated and fragmented forests in peri-urban areas [44,123,124,125,126]. A potential explanation for this could be that threatening landscape processes, such as habitat loss and fragmentation, may reduce the abundance and diversity of the prey species available to dingoes [127,128]. This could trigger a prey-switching response and amplify predation pressure on species that would otherwise be undesirable as prey.

## 5. Conclusions

Carnivores perform complex ecological roles that are vital to maintaining ecosystem health [19,20,23,25,27,30,67]. Control of carnivore populations in natural systems should, therefore, carefully consider the ecological trade-offs of removal and only occur where predation has been shown to substantially threaten livestock enterprises or place populations of threatened species at risk. In NE NSW, high predation rates on pademelons documented by past dietary studies [82,83] sparked concern that dingoes may threaten small macropods in the region. Our study suggests that although dingoes may prey upon some threatened macropods in some subtropical NE NSW forests, they appear to do so sustainably under the prevailing conditions. Nonetheless, it is important to remain cautious as stochastic environmental processes such as drought and fires have the potential to influence prey population demographics and, in turn, predator–prey dynamics [45,129,130,131]. Therefore, our findings should not be interpreted as a reason for complacency. Rather, we advocate for ongoing population monitoring of both carnivores and prey species of concern, as well as the periodic monitoring of carnivore diets. We also encourage future predator dietary studies to incorporate data on prey availability into their design. This approach would enable researchers and managers to better assess and potentially model the population-level impacts of a carnivore’s feeding habits. Furthermore, it would facilitate the timely detection of adverse changes to predator–prey dynamics, allowing for the development of effective conservation and management strategies, should they be necessary.

## Figures and Tables

**Table 1:** Percentage frequency of occurrence (FOC) and percentage volume (Vol) of prey species in dingo scats collected in the Border Ranges and Richmond Range National Parks. Prey categories and category totals are in bold. ‘*n*’ denotes sample size, and * indicates introduced species.

Prey Item		Border Ranges		Richmond Range	
		*n* = 322		*n* = 190	
Common Name	Species Name	FOC (%)	Vol (%)	FOC (%)	Vol (%)
**Small mammals**		**8.1**	**3.2**	**5.3**	**1.8**
Fawn-footed melomys	*Melomys cervinipes*	2.5	0.9	0.5	0.1
House mouse *	*Mus musculus* *			1.6	0.6
Bush rat	*Rattus fuscipes*	3.4	1.7		
Swamp rat	*Rattus lutreolus*	0.3	0.2		
Black rat *	*Rattus rattus* *	1.2	0.2	0.5	0.5
Unidentified rat	*Rattus* sp.	0.3	0.0	1.6	0.1
Sugar glider	*Petaurus breviceps*	0.3	0.2	0.5	0.2
Squirrel glider	*Petaurus norfolcensis*			0.5	0.4
**Medium-sized mammals**		**96.0**	**88.6**	**64.2**	**57.5**
Short-beaked echidna	*Tachyglossus aculeatus*	0.3	0.1	12.1	8.7
Northern brown bandicoot	*Isoodon macrourus*	1.9	1.2	1.6	0.7
Long-nosed bandicoot	*Perameles nasuta*	0.9	0.8		
Unidentified bandicoot	*Isoodon/Perameles* sp.	1.2	0.1	1.1	0.3
Grey-headed flying fox	*Pteropus poliocephalus*	0.3	0.3	0.5	0.0
Common ringtail possum	*Pseudocheirus peregrinus*	53.1	48.3	2.1	1.5
Short-eared possum	*Trichosurus caninus*	24.5	22.9	3.2	2.9
Common brushtail possum	*Trichosurus vulpecula*	10.9	10.1	2.1	1.8
Unidentified possum	*Trichosurus* sp.	4.0	2.4	1.6	1.1
Rufous bettong	*Aepyprymnus rufescens*			0.5	0.5
Red-legged pademelon	*Thylogale stigmatica*	1.2	1.2	23.2	21.4
Red-necked pademelon	*Thylogale thetis*	0.3	0.3	7.4	7.4
Unidentified pademelon	*Thylogale* sp.	1.2	0.3	16.3	11.1
Feral cat *	*Felis catus* *	0.9	0.6		
**Large mammals**				**26.8**	**31.3**
Canid	*Canis* sp.			1.1	1.1
Black-striped wallaby	*Notamacropus dorsalis*			1.1	1.1
Pretty-face wallaby	*Notamacropus parryi*			0.5	0.5
Red-necked wallaby	*Notamacropus rufogriseus*			1.1	1.1
Swamp wallaby	*Wallabia bicolor*			5.8	5.8
Domestic cattle *	*Bos taurus* *			17.4	14.1
**Unidentified macropod**		**0.9**	**0.6**	**8.9**	**7.7**
**Birds**		**18.6**	**4.2**	**12.1**	**5.2**
**Reptiles**		**7.5**	**1.7**	**0.5**	**0.2**
Skink		4.0	1.1		
Varanid				0.5	0.2
Dragon		0.3	0.0		
Snake		3.1	0.6		
**Invertebrates**		**0.9**	**0.0**	**1.1**	**0.0**
**Plant material**		**3.4**	**1.8**	**8.9**	**4.0**

**Table 2:** Results of SIMPER analyses for species and prey categories that contributed most (>10%) to observed dissimilarity between reserve and season sample groups with significant differences (*p* < 0.05). Average dissimilarity indicates the strength of discrepancies in occurrence of species or prey categories between diets. Contribution (%) shows the average contribution that each species or prey category makes to dissimilarity between sample groups. Superscript 1, 2 indicates in which sample group a species or prey category occurs more often. BR—Border Ranges; RR—Richmond Range; S—small mammals; and M—medium-sized mammals.

Sample Group Comparisons	Species/Category	Avg. Dissimilarity	Contribution (%)	Cumulative (%)
BR ^1^ vs. RR ^2^	Ringtail possum	20.46 ^1^	21.19	21.19
	Short-eared possum	10.46 ^1^	10.83	32.01
	Red-legged pademelon	10.13 ^2^	10.48	42.50
Border Ranges				
Summer ^1^ vs. winter ^2^	Bird	12.91 ^1^	45.56	45.56
	Plant matter	4.90 ^1^	17.29	62.85
	S	4.36 ^2^	15.38	78.23
	M	4.12 ^2^	14.54	92.77
Winter ^1^ vs. spring ^2^	Bird	9.53 ^2^	36.73	36.73
	S	5.43 ^1^	20.92	57.65
	Reptile	5.22 ^2^	20.13	77.78
	M	3.06 ^2^	11.79	89.58

**Table 3:** Jacob’s selectivity index (*D*) for potential prey species in the Border Ranges (BR) and Richmond Range (RR). *r*—frequency in dingo diet, *p*—availability, and *n*—average daily detections.

	Potential Prey	*r*	*p*	*D*	*n*
BR	Red-legged pademelon	0.02	0.43	−0.94	131.0
	Long-nosed potoroo	0.00	0.09	−1.00	28.0
	Short-eared brushtail possum	0.27	0.27	0.01	81.5
	Long-nosed bandicoot	0.01	0.15	−0.86	46.0
	Feral cat	0.01	0.06	−0.72	17.5
RR	Red-legged pademelon	0.36	0.28	0.16	138.5
	Red-necked pademelon	0.11	0.11	0.03	52.0
	Long-nosed potoroo	0.00	0.02	−1.00	10.0
	Short-eared brushtail possum	0.04	0.06	−0.23	31.5
	Long-nosed bandicoot	0.00	0.41	−1.00	201.5
	Northern brown bandicoot	0.03	0.09	−0.57	44.0
	Koala	0.00	0.02	−1.00	11.0

## Data Availability

Data can be supplied on reasonable request.
